# Assessing the role of two populations of *Aedes japonicus japonicus* for Zika virus transmission under a constant and a fluctuating temperature regime

**DOI:** 10.1186/s13071-020-04361-2

**Published:** 2020-09-18

**Authors:** Uros Glavinic, Jasmin Varga, Anca Ioana Paslaru, Jeannine Hauri, Paul Torgerson, Francis Schaffner, Eva Veronesi

**Affiliations:** 1 National Centre for Vector Entomology, Institute of Parasitology, Vetsuisse Faculty, University of Zürich, Zürich, Switzerland; 2 Department of Biology, Faculty of Veterinary Medicine, University of Belgrade, Belgrade, Serbia; 3 Section of Epidemiology, Vetsuisse Faculty, University of Zürich, Zürich, Switzerland; 4 Francis Schaffner Consultancy, Lörracherstrasse 50, 4125 Riehen, Switzerland

**Keywords:** *Aedes japonicus*, Fluctuating temperature, Vector competence, Zika virus

## Abstract

**Background:**

Since the huge epidemic of Zika virus (ZIKV) in Brazil in 2015, questions were raised to understand which mosquito species could transmit the virus. *Aedes aegypti* has been described as the main vector. However, other *Aedes* species (e.g. *Ae. albopictus* and *Ae. japonicus*) proven to be competent for other flaviviruses (e.g. West Nile, dengue and yellow fever), have been described as potential vectors for ZIKV under laboratory conditions. One of these, the Asian bush mosquito, *Ae. japonicus*, is widely distributed with high abundances in central-western Europe. In the present study, infection, dissemination and transmission rates of ZIKV (Dak84 strain) in two populations of *Ae. japonicus* from Switzerland (Zürich) and France (Steinbach, Haut-Rhin) were investigated under constant (27 °C) and fluctuating (14–27 °C, mean 23 °C) temperature regimes.

**Results:**

The two populations were each able to transmit ZIKV under both temperature regimes. Infectious virus particles were detected in the saliva of females from both populations, regardless of the incubation temperature regime, from 7 days post-exposure to infectious rabbit blood. The highest amount of plaque forming units (PFU) (400/ml) were recorded 14 days post-oral infection in the Swiss population incubated at a constant temperature. No difference in terms of infection, dissemination and transmission rate were found between mosquito populations. Temperature had no effect on infection rate but the fluctuating temperature regime resulted in higher dissemination rates compared to constant temperature, regardless of the population. Finally, transmission efficiency ranged between 7–23% and 7–10% for the constant temperature and 0–10% and 3–27% under fluctuating temperatures for the Swiss and the French populations, respectively.

**Conclusions:**

To the best of our knowledge, this is the first study confirming vector competence for ZIKV of *Ae. japonicus* originating from Switzerland and France at realistic summer temperatures under laboratory conditions. Considering the continuous spread of this species in the northern part of Europe and its adaptation at cooler temperatures, preventative control measures should be adopted to prevent possible ZIKV epidemics.
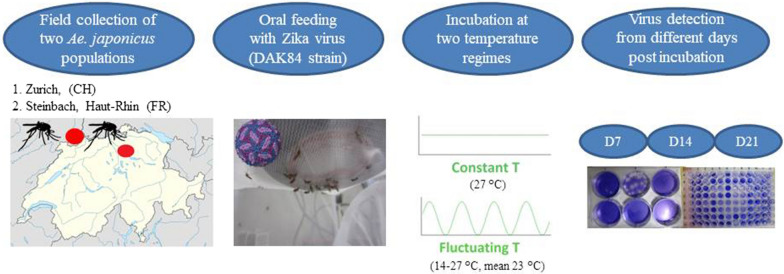

## Background

The first appearance of the invasive bush mosquito *Aedes* (*Hulecoeteomyia*) *japonicus japonicus* (Theobald 1901) (Diptera: Culicidae) in Europe was in 2000 [1] in France. In 2008, *Ae. japonicus* was first detected in Switzerland [2], in the north-west part of the country from where it spread and became widely established with locally high abundances. Recently, the species has established in other European countries [2] such as Austria, Croatia, Germany, Hungary, Italy, Liechtenstein, The Netherlands, Slovenia and Spain [3–8].

According to genetic studies of the populations collected in Europe, it has been postulated that two separate introductions of *Ae. japonicus* resulted in the presence of two genotypes (genotype I and II) [9, 10]. The first genotype includes populations from Belgium and southwestern Germany/Switzerland, eastern Austria/Slovenia and southeastern Germany/northwestern Austria whereas genotype II includes specimens from western and northern Germany only [10, 11].

The role of this species as putative vector of arboviruses such as Eastern equine encephalitis virus (EEEV) [12], La Crosse encephalitis virus (LACV) [13], West Nile virus (WNV) [14, 15], dengue virus (DENV), chikungunya virus (CHIKV) [16] and filarial parasites [17] has been well described. Since the large outbreak of Zika virus (ZIKV) in South America [18] in 2015, huge attention has been given to investigate which mosquito species could be involved in its transmission [19] and how this can vary between vector populations and virus strains [20–23]. Several *Aedes* species have been proven to be capable of transmitting ZIKV [24] including autochthonous and invasive European species (*Ae. albopictus*) [25–27]. Although *Ae. aegypti* has been described as the main vector species for ZIKV transmission [24], recent studies have also shown the potential role of an *Ae. japonicus* population from southwestern Germany in the transmission of a ZIKV strain from Guatemala (Central America) [26].

Our recent findings have confirmed the potential role of a population of *Ae. japonicus* from Switzerland for the transmission of flaviviruses such as WNV, CHIKV and DENV [14, 15]. ZIKV belongs to the same family as WNV and DENV, therefore it was pertinent to investigate the transmission potential of *Ae. japonicus* for ZIKV. The effect of different temperatures on the infection, dissemination and transmission of ZIKV for a German population of *Ae. japonicus*, was also investigated in the work of Jansen et al. [26]. However, although the authors have demonstrated transmission occurring at 27 °C, they used constant temperature regimes and not fluctuating which would have been a more realistic reflect of the natural field conditions of variable temperatures. Consequently, we wished to also investigate if transmission varied according to temperature regime and population of *Ae. japonicus*.

In our study, we have tested one strain of ZIKV from Dakar (DAK84 Senegal, Africa) [27] with populations of *Ae. japonicus* from Switzerland (Zürich) and France (Steinbach, Haut-Rhin). Several vector competence indices from orally infected *Ae. japonicus* females were investigated: infection (virus presence in abdomen-thorax); dissemination (virus presence in head); transmission (virus presence in saliva), and transmission efficiency (females with positive saliva among tested females). Two major aims were addressed: (i) to determine the susceptibility to oral infection with ZIKV of two field-collected *Ae. japonicus* populations; and (ii) to evaluate how fluctuating *vs* constant temperature is affecting the susceptibility to ZIKV infection, dissemination and transmission within these *Ae. japonicus* populations.

## Methods

### Mosquito rearing

Field-collected *Ae. japonicus* were used for this study. Eggs were collected with standard ovitraps baited with germination paper as oviposition substrate [26]. Two different localities were chosen for collection. The first was located in Zürich, Switzerland, with two collection sites, the Schwamendingen cemetery (47°24′5″N, 8°34′28″E) and a private garden close to the University of Zürich - Irchel Campus (47°23′45.3″N, 8°33′04.6″E). The second location was a private garden (47°49′16.02″N, 7°8′59.20″E) in Steinbach (Haut-Rhin, France). Overall, 30 ovitraps were placed in Zürich (10 in the private garden and 20 in the cemetery) and 20 in Steinbach during June-August 2017. Germination papers were changed weekly, stored semi-dry in plastic zip bags for 7 days at room temperature and then placed in plastic trays with 2 l of deionized water. One day post-immersion, hatched larvae were counted, split (400 larvae/tray) and supplemented with yeast tablets (Gayelord Hauser Superlevure, Gayelord Hauser, France) as larval food (2 tablets/tray). If necessary, half a tablet was added few days later. Larvae were incubated at 27 °C with 85% relative humidity (RH) and the adults obtained were kept into polyester cubic netted cages (32.5 × 32.5 × 32.5 cm) (Bugdorm 43030F, MegaViewScienceCo. Ltd., Taichung, Taiwan) under long daylight conditions (16:8 h L:D) including 1 h dusk and 1 h dawn. A 10% sucrose solution was provided daily to the adults as carbohydrate source.

### Mosquito infection

Lyophilized ZIKA virus Dak84 strain, isolated in Dakar (GenBank: KU955592 [27]) (7.57 log_10_ TCID_50_/ml) and provided by Dr Failloux (Institute Pasteur, Paris, France). The virus was re-suspended into 400 µl of distilled water, then mixed (1:3) with washed heparinized rabbit blood (not older than 24 h) obtained from the slaughterhouse of a private company (H. R. Kyburz AG, Dorfstrasse 32, Lupfig, Switzerland) to obtain a final a titer of 7.1 log_10_ TCID_50_/ml. Finally, phagostimulant (ATP at 5 × 10^−3^ M) was added to each blood meal. Seven to 9 days-old females were deprived of sugar 24 h before their exposure to virus-spiked blood as previously described [14]. Briefly, immediately after the preparation of the infectious blood mixture, 3 ml were transferred into a Hemotek feeder (Hemotek Ltd., Lancashire, UK) and covered with a pork intestine membrane fixed with a rubber ring. Mosquitoes were aspirated from the rearing cages and transferred into 500 ml plastic bottles (*c.*60 females/bottle) with the top side covered with a fine net through which the mosquitoes were exposed to the Hemotek feeder. After 20 min of exposure to the infectious blood, mosquitoes were anesthetized by placing the bottles at − 20 °C for about 4–5 min and then transferred onto a Petri dish previously layered with filter paper. The Petri dish was kept on an ice pack the whole time to keep the mosquitoes anesthetized. Fully engorged females were collected and placed into a cardboard box cylinder (12 cm diameter and 15 cm length) covered with nets at both sides which in turn was allocated into a bugdorm cage. Freshly engorged mosquitoes (two females/experiment) were collected immediately after blood-feeding (Day 0) as well as a small aliquot of the infectious inoculum for further analysis. All the engorged females were incubated under two different climatic conditions: (i) constant temperature (27 °C and relative humidity 85%); and (ii) fluctuating temperature (21 ± 7 °C (mean 23 °C) with 45–90% relative humidity), reflecting a typical day in northern Switzerland in mid-summer (www.meteoswiss.admin.ch). The photoperiod for both temperatures was the same as described above. Accordingly, there were four different groups of engorged mosquitoes: Steinbach (i) at constant and (ii) fluctuating temperature; Zürich (iii) at constant and (iv) fluctuating temperature. At different time points (days 7, 14 and 21 post-oral feeding), 30 females from each infection group were collected. Cardboard boxes were placed at -20 °C for 4–5 min to anesthetize the survived mosquitoes after which they were transferred onto a Petri dish with an ice pack as described above and processed for virus detection. From each time point collection we investigated the rate of infection (IR, proportion of females with infected abdomen among tested females), dissemination (DR, proportion of females with infected heads among infected females), transmission (TR, proportion of females with infected saliva among the females with disseminated infection) and transmission efficiency (TE, proportion of females with infectious saliva among all tested females). All feeding, manipulation and incubation of ZIKV infected mosquitoes were carried out at biosafety containment level 3 (BSL3).

### Virus detection in abdomen (infection) and head (dissemination)

After the incubation period, females were dissected for removal of wings and legs with sterile forceps and the rest of the body (head, abdomen and thorax) stored dry in 1.5 ml Eppendorf tubes at − 80 °C until further investigation. Infection and virus dissemination, confirmed by the presence of virus particles in the tissues of abdomen and thorax (body) and heads, respectively, was determined from body parts’ homogenates. A Tissue Lyser® II instrument (Qiagen, Hilden, Germany) was used for homogenization, at 25 Hz for 1 min, followed by 5 min centrifugation at 13,000×*g* at 4 °C as described [14]. Briefly, 300 µl of Eagle’s Minimum Essential Medium (EMEM) (LGC Standard, GmbH, Wesel, Germany) supplemented with 1% antibiotics and fungizone (1000 IU/ml penicillin/streptomycin; 4 µg/ml amphotericin) (Gibco, Thermo Fisher Scientific, Reinach, Switzerland) (EMEM complete), 2% fetal bovine serum (FBS), and one stainless steel bead (3 mm diameter) were added to each tube containing either the head or the body of the individual mosquitoes. 96-well plates layered with Vero cells (30,000 cells/100 µl/well) and EMEM complete supplemented with 10% FCS were prepared 1 day prior to Vero cell infection, and incubated at 37 °C with 5% CO_2._ When cells were 75–80% confluent, medium was removed and 100 µl of serial dilutions of body part homogenates (neat, 1:10 and 1:100) was inoculated into the monolayer of Vero cells. After the incubation period, cells were stained with a crystal violet solution (0.2% of crystal violet, 10% formaldehyde and 10% ethanol) in order to identify positive wells. Briefly, 2 ml of the crystal violet solution was added to each well followed by 30 min incubation at room temperature after which the wells were washed twice with deionized water and the presence of viral particle assessed by detection of cytopathic effects (CPE) under a microscope. The whole body of day 0 females was also homogenized and titrated to confirm virus titre. Briefly, a serial dilution (1:10, 1:100, 1:1000, 1:10,000 and 1:100,000) was loaded on a 96-well plate layered with Vero cells as described above for the abdomen, thorax and heads. After a 7-day incubation, cells were checked and titre calculated based on the presence of plaques.

### Virus detection and quantification in saliva (transmission)

For saliva collection, after the removal of wings and legs from each survived individual, the proboscis was inserted into 20 µl pipette tips filled with 5 µl of FBS. After 30 min salivation, the 5 µl of FBS with collected saliva were transferred into 1.5 ml Eppendorf tubes containing 45 µl of EMEM complete, giving a final volume of 50 µl. Saliva was held on ice until all the samples were collected and then frozen at − 80 °C.

The quantification of infectious virus particles was determined by a plaque forming unit assay and expressed as PFU/saliva. Briefly, 6-well plates layered with 75–80% confluent Vero cells (800,000 cells/2 ml/well) were inoculated with EMEM complete supplemented with 10% FBS 24 h before incubation with saliva. For the infection, the medium was removed from each well and 265 µl of EMEM complete supplemented with 2% FBS was added to each well, followed by 35 µl of the saliva sample giving a total volume of 300 µl/well. The remaining 15 µl of the saliva samples were kept at − 80 °C as a back-up. After 1 h incubation at 37 °C, 4 ml of a 0.5% agarose solution (UltraPure™ Agarose, Invitrogen Life Technologies, Renfrew, UK) in EMEM complete was added to each well without the removal of the inoculum, and all plates incubated at 37 °C with 5% CO_2_. At day 7 post-incubation, the agarose gel was removed and cells were stained by adding 2 ml of crystal violet solution per well. After 30 min incubation at room temperature, cells were rinsed with water and plaques enumerated according to the volume of the sample tested and expressed as PFU/saliva.

### Statistical analysis

Differences in the rates of infection, dissemination, and transmission between the two temperature conditions (fluctuating, 21 ± 7 °C; and constant, 27 °C) and the two populations of *Ae. japonicus* were analysed by logistic regression.

There were therefore three logistic regression models. The first considered infection of mosquitoes as a binomial dependent variable (virus detected in the abdomen as infected or no virus detected as not infected). The independent variables were site (categorical variable, France or Zürich), time (continuous variable *n* days post-oral feeding) and temperature (categorical variable, constant or fluctuating).

The second logistic regression model considered only those mosquitoes in which infection was proven. In this case dissemination was the dependent variable (virus detected in the head as disseminated or not disseminated).

The third logistic regression model only considered those mosquitoes in which there was dissemination of virus. The detection of virus in the saliva (transmission) was the dependent binomial variable (positive or negative). The same independent variables for the first logistic regression model were used in the second and third regression model. Interactions between independent variables were also analysed. All proportions (infected/not infected, disseminated/not disseminated, infectious/not infectious) are reported with exact 95% binomial confidence intervals. The association of viral growth (copy number) with time or temperature was analysed using a negative binomial generalized linear model (GLM) with a log link function. This assumed that copy number was an integer dependent variable, with a minimum value of zero that was overdispersed. Hence the negative binomial model was considered the most appropriate model for analysis. For all regression models, backward stepwise elimination was used to remove non-significant variables in the model. All possible interactions in the models were also examined. Regression models were also undertaken taking data from the two sites in separate models for an insight into variables that may only have an association at one of the two study sites. Significant variables remaining within models are reported as the *P*-value and corresponding *Z-*statistic. All analyses were undertaken in R [16].

## Results

### Mosquito infection

Overall, 2460 *Ae. japonicus* females were exposed to blood spiked with the ZIKV Dak84 strain, with a final titer of 7.0 log_10_ TCID_50_/ml, and a total of 739 individuals (450 from Zürich and 289 from Steinbach) were successfully engorged (feeding rates 8–33%).

### Virus detection in abdomen (infection) and head (dissemination)

Infection rates (IR) (Table 1), based on the cytopathic effect (CPE) on Vero cells, for the Zürich population of *Ae. japonicus* incubated at a constant temperature, ranged between 83–93% (Additional file 1: Figure S1) whereas the rates for mosquitoes incubated at fluctuating temperatures were 17–77%. Similarly, the infection rate of the Steinbach population ranged between 67–100% at a constant temperature, and 47–73% at fluctuating temperature (Table 2) (Additional file 1: Figure S1). Logistic regression analyses showed that there were no significant differences in the rates of infection between the two populations of *Ae. japonicus* and in the infection rate according to the incubation temperature (fluctuating *vs* constant temperature) and the length of incubation post oral infection (day 7, 14 or 21).

**Table 1:** Proportion (rates) of *Aedes japonicus* females from Zürich (Switzerland), positive to ZIKV in the abdomen (infection), head (dissemination) and saliva (transmission) after feeding on ZIKV-spiked blood

DPI	IR (%)				DR (%)				TR (%)				TE (%)			
	C	95% CI	F	95% CI	C	95% CI	F	95% CI	C	95% CI	F	95% CI	C	95% CI	F	95% CI
7	25/30 (83)	65–94	14/30 (47)	28–66	10/25 (40)	21–61	10/14 (71)	42–92	7/10 (70)	35–93	3/10 (30)	7–65	7/30 (23)	1–42	3/30 (10)	2–26
14	28/30 (93)	78–99	5/30 (17)	6–35	9/28 (32)	16–52	3/5 (60)	15–95	6/9 (67)	30–92	0/3 (0)	0–7	6/30 (20)	8–38	0/30 (0)	0–11
21	26/30 (87)	69–96%	23/30 (77)	58–90	3/26 (12)	2–30	6/23 (26)	10–48	2/3 (67)	9–99	3/6 (50)	12–88	2/30 (7)	0.8–22	3/30 (10)	2–26

*Abbreviations*: DPI, days post-oral feeding; C, constant temperature (27 °C); F, fluctuating temperature (21 ± 7 °C); IR, infection rate (proportion of survived mosquitoes containing infectious virus particles in the abdomen); DR, dissemination rate (proportion of survived mosquitoes containing infectious virus particles in the head among infected mosquitoes); TR, transmission rate (proportion of survived mosquitoes containing infectious virus particles in the saliva among those with disseminated infection); TE, transmission efficiency (proportion of females with positive saliva among all tested females); 95% CI, 95% confidence interval

**Table 2:** Proportion (rates) of *Aedes japonicus* females from Steinbach (France) positive to ZIKV in the abdomen (infection), head (dissemination) and saliva (transmission) after feeding on ZIKV-spiked blood

DPI	IR (%)				DR (%)				TR (%)				TE (%)			
	C	95% CI	F	95% CI	C	95% CI	F	95% CI	C	95% CI	F	95% CI	C	95% CI	F	95% CI
7	20/30 (67)	47–83	22/30 (73)	54–88	8/2 (40)	19–64	7/22 (32)	14–55	2/8 (25)	3–65	3/7 (43)	10–81	2/30 (7)	0.8–22	3/30 (10)	2–26
14	30/30 (100)	88–100	18/30 (60)	40–77	6/60 (20)	8–38	11/18 (61)	36–83	3/6 (50)	12–88	8/11 (73)	40–94	3/30 (10)	2–26	8/30 (27)	12–46
21	20/30 (67)	47–83	14/30 (47)	28–66	5/20 (25)	9–49	9/14 (64)	35–87	2/5 (40)	5–85	1/9 (11)	0.2–48	2/30 (7)	0.8–22	1/30 (3)	0.08–17

*Abbreviations*: DPI, days post-oral feeding; C, constant temperature (27 °C); F, fluctuating temperature (21 ± 7 °C); IR, infection rate (proportion of survived mosquitoes containing infectious virus particles in the abdomen); DR, dissemination rate (proportion of survived mosquitoes containing infectious virus particles in the head among infected mosquitoes); TR, transmission rate (proportion of survived mosquitoes containing infectious virus particles in the saliva among those with disseminated infection); TE, transmission efficiency (proportion of females with positive saliva among all tested females); 95% CI, 95% confidence interval

Within the Zürich population (*n *= 121), dissemination rate (DR) was higher (*Z *= 2.339, *df *= 118, *P *= 0.019) when females were incubated at a fluctuating temperature compared to constant temperature, ranging between 26–71% and 12–40% (Additional file 1: Figure S1), respectively, whereas DR decreased over time (length of incubation 7, 14 and 21 days) (*Z *= − 3.409, *df *= 118, *P *= 0.0067) regardless of the incubation temperature regimes. Within the Steinbach population (*n *= 122) of *Ae. japonicus* there was an interaction between fluctuating temperature and time with an increased dissemination rate over time under fluctuating temperature conditions (*Z *= 2.612, *df *= 118, *P *= 0.009) (32–64%) but not if incubated at a constant temperature (20–40%).

Viral titer among females collected straight after oral infection (day 0 females) ranged between 4.25–5.25 log_10_ PFU/ml.

### Virus detection and quantification in the saliva (transmission)

Infectious virus particles were detected in the saliva of females from both populations incubated at either constant or fluctuating temperature regimes. No statistical differences were found in the transmission rate between the two populations and between the two temperature regimes. However, transmission rates for mosquitoes which originated from Steinbach (*n *= 122) and incubated at a fluctuating temperature were significantly higher at 14 days post-incubation compared to those after 21 days (*Z *= − 2.432, *df *= 71, *P *= 0.015).

The transmission rate (TR) varied between 67–70% and 30–50% in the Zürich population and 25–50% and 11–73% rate (Additional file 1: Figure S1) for the Steinbach population at constant and fluctuating temperature, respectively (Table 1). Positive saliva samples were observed already at day 7 post-oral infection regardless of incubation conditions and origin of the population.

**Fig. 1:**
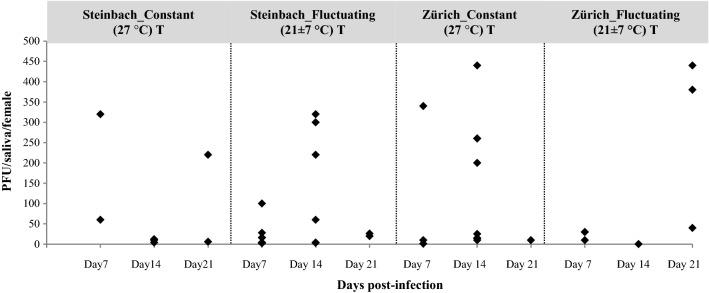
Viral loads of females orally fed with ZIKV-spiked blood. Data are given as PFU/female according to their origin (Zürich and Steinbach) and temperature condition, constant (27 °C) *vs* fluctuating (21 ± 7 °C) for an incubation period of 7, 14 and 21 days

No significant differences were observed for the saliva viral load (number of infectious virus particles/female) according to incubation temperature regime and time in both populations (Fig. 1). There was no statistical evidence for differences in transmission efficiency (TE) (Table 1) between the Zürich population (7–23%) incubated at constant temperature (27 °C) compared to the Steinbach population (7–10%) incubated at the same temperature (Additional file 1: Figure S1). Likewise, there was no statistical evidence for differences between the two populations in their TE at fluctuating temperatures. As shown in Fig. 1, saliva titres from disseminated females ranged from 1.4 to 440 PFU/saliva, with the maximum titre recorded for one *Ae. japonicus* female originated from Zürich and incubated for 14 days at a constant temperature.

## Discussion

To the best of our knowledge, this is the first study exploring the vector competence of *Ae. japonicus* for ZIKV under a fluctuating temperature regime [24]. Our results confirm the potential role of *Ae. japonicus* for ZIKV transmission under laboratory conditions. Moreover, higher dissemination rates were observed for the population from Zürich when incubated under a fluctuating temperature regime (21 ± 7 °C).

Previous studies investigating mosquito vector competence for several pathogens have been carried out under constant temperature regimes, testing several temperature sets to understand the lower degree threshold for virus infections, dissemination and transmission [26, 28–33]. The main concern of running vector competence studies using a constant temperature for incubation of infected arthropods is that this is not mimicking the real situation in the field where mosquitoes are constantly exposed to fluctuating temperatures. Since arthropods cannot thermoregulate, environmental temperatures play a crucial role on both the ecology and the vectorial capacity of arthropod vectors, affecting parameters such as mortality rate, length of the gonotrophic cycle, biting rate, etc.

Temperature has also a large impact on the amplification of pathogens inside the arthropod body. The length of time required for a pathogen to reach the salivary gland after the intake of an infectious blood meal, the so-called extrinsic incubation period (EIP), is indeed largely regulated by temperature [32, 33]. A correlation between the EIP and temperature is described, with higher temperatures increasing the speed of viral replication, infection and transmission (shorter EIP). Lower temperatures increase the length of the EIP, with an implication for the efficiency of being a vector [30, 34]. However, most of the time the correlation between EIP and temperature has been demonstrated under laboratory conditions where commonly constant temperatures were applied and not fluctuating ones. Some studies on *Culex* species infected with WNV have shown that fluctuating temperature regime have no impact on the rate of the EIP compared to constant mean daily temperatures [34, 35]. However, recent papers have actually demonstrated that even under a fluctuating temperature regime, vector competence can be influenced by a large (20 °C) or moderate (10 °C) diurnal temperature range (DTR) is [36]. Lambrechts et al. [36] described a lower midgut infection at large DTR compared to midgut infection observed under moderate DTR for *Ae. aegypti* infected with two DENV serotypes, despite average temperatures being the same (26 °C).

In our study, a fluctuating temperature regime with a DTR of 14 °C and a mean of 23 °C was shown to increase virus dissemination rate compared to a constant temperature of 27 °C among ZIKV-infected *Ae. japonicus* regardless of the population used. The decrease in the dissemination rate over time recorded here in both populations (except for the French population when incubated at a fluctuating temperature) was also described for other vectors when infected with Zika or dengue virus [37, 38]. We could speculate that dissemination barriers present in the haemocoel of these mosquitoes are very efficient, though to confirm this further studies should be carried out.

Overall, no significant differences for infection, transmission and dissemination were observed between the two populations, although they both show the potential to transmit the ZIKV strain tested here. Further investigations on the genetic make-up of the two populations are here suggested to better clarify their relatedness. These results are in contrast with a recent study investigating ZIKV transmission for a population of *Ae. japonicus* from Germany, where three temperatures were tested (constant 21, 24 and 27 °C) for an incubation period of 14 days. In this study [26], transmission was observed only at 27 °C. Dissemination was detected at 24 and 27 °C with the highest rate at 24 °C but a higher virus titer at 27° C. Midgut infection was recorded at all the three temperatures with the lowest rate at 21 °C.

Although in our study we have recorded transmission of ZIKV at a mean temperature of 23 °C, we can infer that, in the German population, other parameters must have been involved in preventing dissemination of the virus within the mosquito. Indeed, it is not the first time that the Swiss population (Zürich) of *Ae. japonicus* has been shown to be more competent than a population from Germany (Stuttgart) [11] when exposed to the same virus (WNV) [14, 15, 39]. In this previous study [39], the population from Germany was completely negative for WNV infection after 14 days post-oral exposure to infectious blood. On the other hand, another population of the same species originating from the USA [40] was found capable of WNV transmission under laboratory conditions when incubated at 26 °C. The authors have speculated that differences in genetic background between *Ae. japonicus* populations from Germany and North America are most likely responsible for the differences in vector competence for WNV [39]. Indeed, vector competence is controlled by several factors including the genetic background of the mosquito species which can vary between different populations and the pathogen strain used [41]. We can consider that the Swiss, the French and the German populations of *Ae. japonicus* were derived from the same introduction since they were collected from a continuous colonized area, probably originating from Switzerland [2]. Genetic analyses of German and Swiss populations of *Ae. japonicus* have described the Swiss population as more closely related to the population from southern Germany (Waldshut-Tiengen, Baden-Württemberg) rather than to the northern population from Bonn [39]. Thus, the different susceptibility to virus transmission between the Swiss and the southern Germany (Stuttgart) populations might not be a genetically driven effect. Clearly, more investigations are required to assess the influence of the type of assay used, general methodologies applied during the experiments and the strain of ZIKV used for oral infection. In our study we have used a highly virulent ZIKV strain (DAK84, GenBank: KU955592) [27, 42] from Senegal, very closely related to another strain (GenBank: KU955591) originating from the same area (Dakar, Senegal) [41, 42] that has previously demonstrated to be successfully transmitted by *Ae. aegypti* under laboratory conditions [43]. The study by Jansen et al. [26] was carried out using a strain from Guatemala (Mexico) (GenBank: KU870645) [43] closely related to another ZIKV strain (GenBank: KX247632) [44] which has already been shown to be not very infectious for mosquitoes, since *Ae. aegypti* originating from Brazil and USA orally exposed to this strain were negative in the saliva [24, 45]. However, a very recent paper has shown efficient transmission among *Ae. aegypti* infected with another strain of ZIKV isolated from field-infected mosquitoes originating from Mexico although no sequencing of this strain was carried out [46].

As we have demonstrated here, the pathogen-vector interaction and consequences for virus transmission is a very complex aspect that needs to be considered carefully when assessing vector competence of a mosquito species. The data here presented were produced using mosquitoes infected and handled under laboratory conditions. When assessing vector competence of mosquito species in the transmission of a specific pathogen in the field, other factors (e.g. vector abundance, longevity, biting rate and dispersal) influencing the efficiency of pathogen transmission should also being taken in consideration.

In the present study, we have shown that two populations of *Ae. japonicus* from the western part of Europe (Switzerland and France) are potentially capable of transmitting ZIKV at realistic fluctuating temperatures between 14 and 27 °C with a mean of 23 °C suggesting that there is a risk for ZIKV transmission in these areas and that preventative measures to control the spread and suppress populations of the invasive *Ae. japonicus* should be implemented.

## Conclusions

Our study confirms that, under laboratory conditions, both investigated populations of *Ae. japonicus* could potentially transmit Zika virus under a central European summer temperature regime. Although no differences were detected in infection, dissemination and transmission rates among the two populations for the two temperature regimes, we proved that dissemination increases when mosquitoes are incubated under a fluctuating temperature compared to constant one. More attention should be given to the strain of virus used and the population of mosquitoes exposed to these strains in order to evaluate the impact that the genetic make-up of both the virus and the vectors can have on vector competence, and the consequent implications for the onset of ZIKV epidemics in Europe.

## Supplementary information

**Additional file 1: Figure S1.** Rates of infection, dissemination and transmission for two populations (Zürich and Steinbach) of *Ae. japonicus* incubated at two different temperatures (constant 27 °C and fluctuating 21 ± 7 °C).

## Data Availability

The datasets used and/or analyzed during the present study are available from the corresponding author upon reasonable request. All data generated or analysed during this study are included in this published articles and its additional file.

## Abbreviations

WNV: West Nile virus
EEEV: Eastern equine encephalitis virus
LCV: La Cross virus
DENV: Dengue virus
CHIKV: Chikungunya virus
ZIKV: Zika virus
Vero: Vero cells, African green monkey kidney cells
EMEM: Eagle’s minimum essential medium
FCS: Foetal calf serum
CPE: Cytopathic effect
TCID_50_/ml: 50% tissue culture infectious dose
IR: Infection rate
DR: Dissemination rate
TR: Transmission rate
Dpi: Days post-infection
BSL3: Biosafety security level 3
LASC: Laboratory Animal Service Centre
PFU: Plaque-forming unit
GLM: Generalized linear model
