# Supplementary material for: Assessing the role of two populations of Aedes japonicus japonicus for Zika virus transmission under a constant and a fluctuating temperature regime
Source: Parasit Vectors. 2020 Sep 18;13:479. doi: 10.1186/s13071-020-04361-2 (PMC7501641; doi:10.1186/s13071-020-04361-2)

Additional file 1: Figure S1. Rates of infection, dissemination and transmission for two populations (Zürich, Switzerland, and Steinbach, France) of *Aedes japonicus* incubated at two different temperature regimes (constant 27 °C and fluctuating 21±7 °C). Error bars represent the 95% confidence intervals.

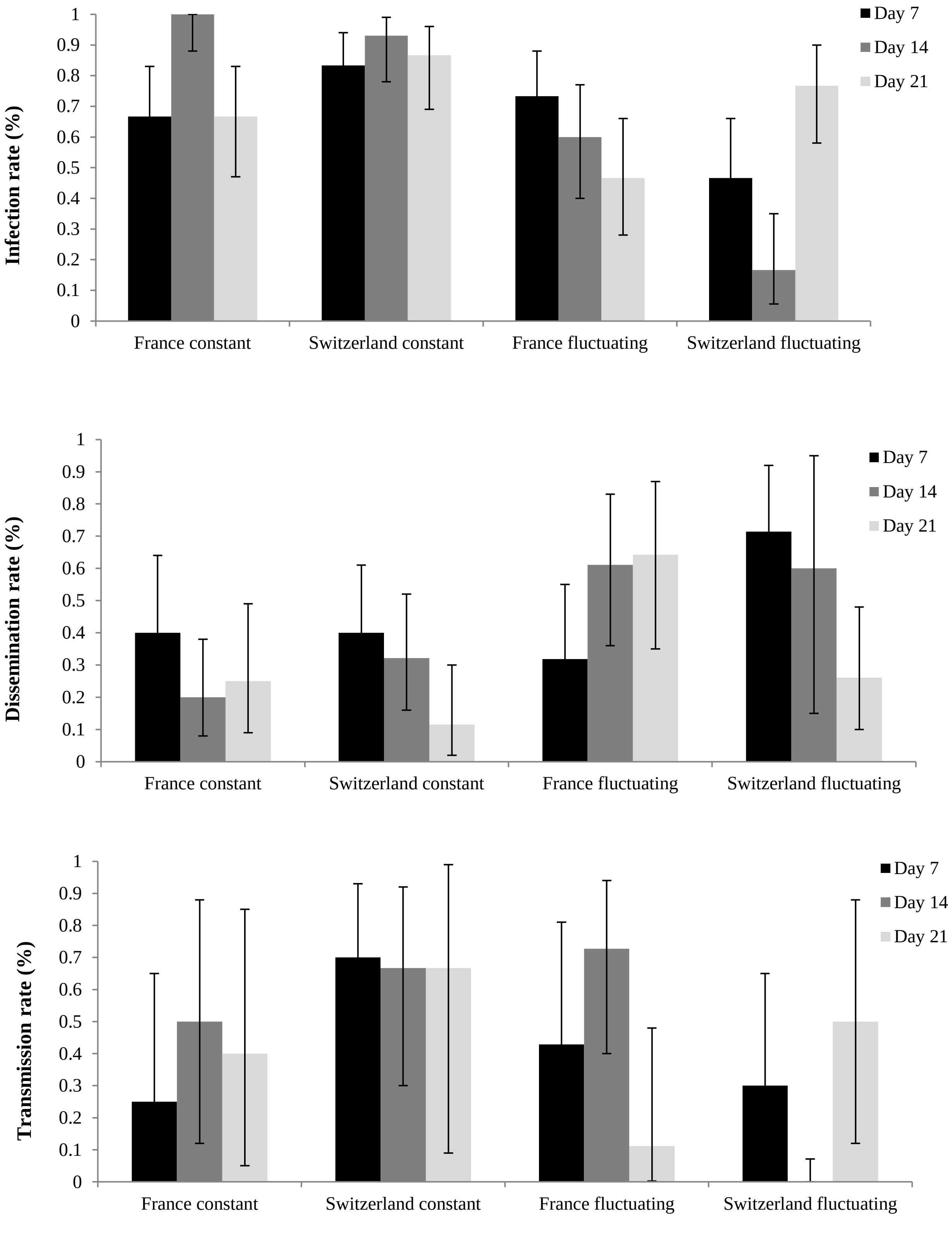

Supplement: Supplementary file 1 — Additional file 1: Figure S1. Rates of infection, dissemination and transmission for two populations (Zürich and Steinbach) of Ae. japonicus incubated at two different temperatures (constant 27 °C and fluctuating 21 ± 7 °C). [file 13071_2020_4361_MOESM1_ESM.pdf]
